# Contrast-Enhanced Ultrasonography Features of Breast Malignancies with Different Sizes: Correlation with Prognostic Factors

**DOI:** 10.1155/2015/613831

**Published:** 2015-12-31

**Authors:** Li-Xia Zhao, Hui Liu, Qing Wei, Guang Xu, Jian Wu, Hui-Xiong Xu, Rong Wu, Huan Pu

**Affiliations:** ^1^Department of Medical Ultrasound, Shanghai Tenth People's Hospital, Tongji University School of Medicine, Shanghai 200072, China; ^2^Ultrasound Research and Education Institute, Tongji University School of Medicine, Shanghai 200072, China; ^3^Department of Pathology, Shanghai Tenth People's Hospital, Tongji University School of Medicine, Shanghai 200072, China

## Abstract

This study was to investigate the correlation between contrast-enhanced ultrasonography (CEUS) characteristics with prognostic factors in breast cancers with different sizes. A retrospective analysis of CEUS characteristics of 104 pathologically proven malignant lesions from 104 women was conducted. Lesions were divided into two groups according to their size measured by US (Group 1: maximum diameter ≤20 mm; Group 2: maximum diameter >20 mm). Features including enhancement degree, order and pattern, enlargement of the enhancement area, and penetrating vessels on CEUS were evaluated. Pathologic prognostic factors, including estrogen and progesterone receptor status, and the expression of c-erb-B2, p53, Ki-67, and VEGF were assessed. Comparison of enhancement pattern parameters between Group 1 and Group 2 showed statistically significant differences (*P* < 0.0001). A significant correlation was found between enlargement of the enhancement area and ER positivity in Group 1 (*P* = 0.032). In Group 2 the absence of penetrating vessels was significantly associated with VEGF negativity (*P* = 0.022) and ER negativity (*P* = 0.022). Centripetal enhancement reflected VEGF negativity (*P* = 0.033) in lesions with diameter >20 mm. Thus, breast cancers with different sizes show different CEUS features; small breast cancers show homogeneous enhancement pattern while cancers with diameter >20 mm show homogeneous enhancement pattern. Some CEUS characteristics of differently sized breast cancers could be correlated with prognostic factors, which may be useful in prognosis assessment.

## 1. Introduction

Contrast-enhanced ultrasonography (CEUS) is becoming an increasingly popular imaging tool in diagnosing breast cancer and can be performed to differentiate between benign and malignant breast lesions [1–3]. Recently, the correlation between CEUS enhancement features in breast cancers with differing prognostic factors has become a focus of intensive research [4, 5]. Previous studies have reported that the enhancement patterns and parameters of breast cancers on CUES, as a noninvasive method, could be used to predict prognosis and to identify highly aggressive breast cancers.

The ultrasound contrast agent (microbubbles) is a blood pool agent and can be used to display the imaging of microvessels. Angiogenesis is crucial for breast cancer growth, invasiveness, and metastasis and is closely related to prognosis [6, 7]. Several factors, such as larger tumor size, shorter doubling time, and higher histologic grade, are also closely related to prognosis [8]. CEUS permits the imaging of capillaries and thus is able to provide evidence toward the identification of benign and malignant breast tumors.

However, regarding the breast cancer with different sizes, its vascular structures, density, and contortion are various. Whether CEUS enhancement features also vary with tumor size remains unclear, and their potential relationship to prognostic variables is also open to debate. Answering these questions would provide a valuable contribution to the diagnosis and prognostic assessment of breast cancer and would permit the rational design of treatment strategies at different stages of the disease; to the best of our knowledge, there is little in the current literatures on this topic. Therefore, the purpose of this study was to investigate the correlation between CEUS performance and prognostic factors in breast lesions of various sizes.

## 2. Materials and Methods

### 2.1. Patients

This retrospective study was approved by the local institutional ethics committee. Informed consent was obtained from all individual participants included in the study. Between August 2012 and July 2014, 131 patients with 133 suspicious malignant breast lesions undergoing CEUS prior to surgical management were enrolled in this study. The inclusion criteria were as follows: (1) suspicious breast lesions classified with the Breast Imaging Reporting and Data System of the American College of Radiology schema as categories 3–5, detected by conventional US or mammography; (2) CEUS examination of lesions before surgery; and (3) pathological examination of lesions after surgical resection, with all relevant prognostic indicators tested by immunohistochemical staining. Those in whom CEUS was contraindicated, as well as pregnant or breastfeeding patients, and those treated with neoadjuvant chemotherapy were all excluded from the study. All benign breast tumors pathologically proven after resection were also excluded.

Among the selected patients, 26 had benign lesions and were excluded from the study. One patient in whom CEUS clips were unsatisfactory due to the excessive influence of respiration movement was also excluded. Two patients with multiple lesions, only the most suspicious lesion meeting the inclusion criteria, were evaluated. Finally, a total of 104 lesions (from 104 patients of mean age of 57.31 ± 10.34 years; range: 33–80 years) were included in the study group.

### 2.2. US Examination

Conventional gray-scale US imaging was performed with the Logiq E9 US system (GE Co., Milwaukee, WI, USA) equipped with a 4–9 MHz linear transducer. All ultrasound examinations were performed by one experienced ultrasound physician with 10 years of experience in breast US and 3 years of experience in breast CEUS. All patients were scanned in the supine position. Firstly, gray-scale and color Doppler US were performed to scan the breast lesion and to observe US features such as the size, position, echogenicity, calcification, intralesional vascularity, and the status of axillary lymph nodes. The maximum plane which included the whole lesion and its surrounding normal tissue was selected for CEUS. Then, the US system was then switched to contrast mode for the CEUS scan. The machine parameters were adjusted as follows: mechanical index, <0.1; gain, 100–120 dB. The parameters remained the same during the examination. SonoVue (Bracco, SpA, Milan, Italy) was used as a contrast agent. 5 mL of sterile normal saline solution was injected into the vial for preparation of the contrast agent before administration; then 2.4 mL of SonoVue was injected via the antecubital vein in a bolus fashion; continuous imaging was performed immediately following this and lasted for 3 minutes. During the examination, the selected plane remained unchanged, with the probe stabilized manually. All US images and video clips were stored on a hard disk within the US machine for subsequent analysis.

### 2.3. Image Analysis

All US data were assessed by two ultrasound physicians with 3 and 2 years of experience in breast CEUS, respectively, who were blinded to the patients' clinical data. If disagreement occurred, the third radiologist with 6 years of experience in breast contrast-enhanced US reviewed the image to reach an agreement. The assessment of the CEUS imaging was based on our clinical experience and the previous studies in the literatures [4].

The lesions were classified into two groups according to their sizes. Group 1 had a maximum diameter ≤20 mm, while Group 2 had a maximum diameter >20 mm. The enhancement degree was classified as hyper-, iso-, or hypoenhancement, assessed relative to the surrounding normal breast tissue at the peak time. With regard to enhancement order, centripetal enhancement was defined as enhancement originating from the periphery of the lesion and developing centripetally, while centrifugal enhancement described enhancement originating from the center of the lesion and developing centrifugally. We defined diffuse enhancement as that showing no order across the lesion. Enhancement pattern was defined as homogeneous when it uniformly and diffusely enhanced the lesion and heterogeneous when this was not the case. Perfusion defect and peripheral enhancement were also included in the latter group. Peripheral enhancement was defined as enhancement occurring exclusively or predominantly at the periphery of the lesion. Assessment of enlargement of the enhancement area involved a comparison between the largest tumorous area of the lesion at the peak time on CEUS and the corresponding tumorous area on gray-scale US; all lesions were classified as having either enlargement or nonenlargement on CEUS. Finally, the presence or absence of penetrating vessels was assessed in the maximum plane including the whole lesion and its surrounding normal tissue on CEUS.

### 2.4. Histopathological Analysis

All surgical samples were analyzed by two pathologists, both of whom have more than 10 years of experience in breast pathologic analysis. The histological grades were assessed according to the Elston-Ellis grading system [9]. Immunohistochemical staining was used to assess the following prognostic indicators: VEGF (MAB293; R&D System, Minneapolis, MN, USA); ER (M0241 [clone 1 D5], Changdao, Shanghai, China); PR (R0448 [clone 1 A6], Changdao, Shanghai, China); c-erb-B2 (A0485, Dako, Glostrup, Denmark); and p53 (M7001 [clone DO7], Doko). For VEGF, the presence of more than 10% of cells showing staining was considered as positive. ER and PR were categorized as negative (<10%) and positive (>10%) in accordance with recent studies [10, 11]. Ki67 status was expressed in terms of the percentage of positive cells, with a threshold of 20% drawn for positivity [4, 12]. For c-erb-B2, immunohistologically Score 3 was positive, for Score 2 results need performing FISH testing [13]. For p53, nuclear staining defined positive expression.

### 2.5. Statistical Analysis

Continuous data were expressed as mean ± standard deviations. Pearson *χ* ^2^ test was used to explore the association between breast lesion size and CEUS enhancement. Correlation of CEUS features with prognostic factors was analyzed using the Pearson *χ* ^2^ test and logistic regression analysis. The level of statistical significance was set at *P* < 0.05. SPSS software (version 16.0, SPSS Inc., Chicago, IL, USA) was used to perform the statistical analysis.

## 3. Results

### 3.1. Histopathological Analysis

The 104 primary breast cancers comprised 93 (89.42%) invasive ductal carcinomas, three (2.88%) ductal carcinomas in situ, two (1.92%) invasive lobular carcinomas, two (1.92%) mucinous carcinomas, three (2.88%) intraductal papillary carcinomas, and one (0.96%) metaplastic carcinoma. According to the Elston-Ellis grading system, the 93 invasive ductal carcinomas included two (2.15%) grade I tumors, 60 (64.52%) grade II tumors, and 31 (31.33%) grade III tumors. The mean size of the breast cancers was 22.51 ± 11.05 mm (5.0–74.0 mm) including 47 small cancers (≤20 mm in diameter; average: 13.64 ± 3.68 mm) and 57 large cancers (>20 mm in diameter; average: 30.77 ± 9.63 mm). The 104 malignant lesions included 69 (66.34%) that were ER positive, 64 (61.54%) that were PR positive, 70 (67.30%) that were Ki-67 positive, 81 (77.88%) that were p53 positive, 53 (50.96%) that were c-erb-B2 positive, and 54 (51.92%) that were VEGF positive.

### 3.2. CEUS Features of Differently Sized Breast Cancers

Comparison of enhancement pattern parameters between Group 1 and Group 2 tumors showed statistically significant differences (*P* < 0.001) (Table 1). However, the differences in the enhancement degree, enhancement order, presence of penetrating vessels, and enlargement of the enhancement area between the two groups were not statistically significant (*P* > 0.05). The distribution of the CEUS enhancement features and the prognostic factors is shown in Table 2.

### 3.3. Correlation of CEUS Features with Prognostic Factors

The association between the CEUS features of differently sized breast cancers and each prognostic factor is summarized in Table 3. In Group 1, centripetal enhancement and enlargement of the enhancement area were correlated with prognostic factors (*P* < 0.05). The absence of penetrating vessels was seen in tumors with ER and VEGF negative expression in Group 2 (*P* < 0.05). Centripetal enhancement showed significant correlation with VEGF negativity, and hyperenhancement was common in tumors with positive c-erb-B2 expression in Group 2 (*P* < 0.05).

### 3.4. Logistic Regression Analysis

**Figure 1:**
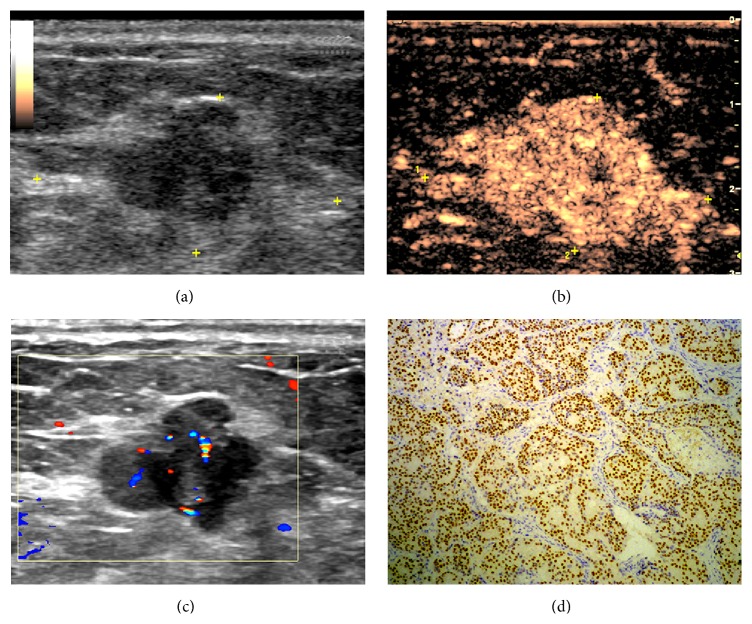
Images in a 70-year-old woman with a 13 mm, grade II invasive ductal carcinoma. (a) Gray-scale US image shows an irregular hypoechoic lesion. (b) Color Doppler US image shows rod-shaped blood flow. (c) CEUS image obtained 23 seconds after contrast agent injection reveals homogeneous hyperenhancement and enlargement of the affected area. (d) Histopathologic analysis demonstrates positive ER expression (original magnification, ×100).

**Figure 2:**
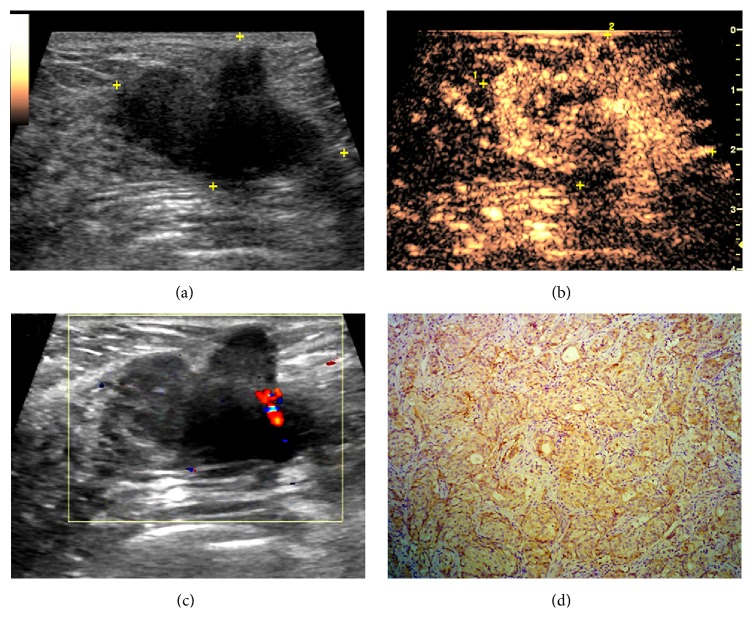
Images in a 60-year-old woman with a 27 mm, grade II invasive ductal carcinoma. (a) Gray-scale image shows an irregular hypoechoic lesion. (b) Color Doppler US image shows dot blood flow. (c) CEUS image obtained 25 seconds after contrast agent injection reveals heterogeneous hyperenhanced mass with local perfusion defects. (d) Histopathologic analysis is negative for VEGF expression (original magnification, ×100).

The results of logistic regression analysis are summarized in Table 4. For Group 1, enlargement of the enhancement area was the best discriminatory criterion for prognosis (Figure 1). For tumors > 20 mm in diameter, the absence of penetrating vessels correlated with VEGF and ER negativity and it may therefore be useful in predicting prognosis. Hyperenhancement may be useful in reflecting positive c-erb-B2 expression in Group 2, while centripetal enhancement was often found in VEGF negative lesions of maximum diameter >20 mm (Figure 2).

## 4. Discussion

At present, tumor prognostic factors can be assessed only in histologic specimens. VEGF plays an important role in stimulating vascular endothelial cell growth and increasing microvessel permeability [6]. Expression of VEGF and its receptor in breast cancer is associated with prognostic factors such as large tumor size and high histologic grade [14–16]. VEGF is not only an independent prognostic indicator for breast malignances but also a possible target for different therapeutic approaches [17]. Expression of the ER and PR also has a close relationship with breast tumor prognosis and the utility of hormonal therapy. Low expression of ER and PR suggests highly aggressive tumors with worse prognosis and poor response to endocrine therapy [18, 19]. The expression of the protooncogene c-erb-B2 can also predict pathogenesis and progress in breast cancer, and its overexpression is closely related to distant metastasis [20]. Meanwhile, Ki-67 is significantly correlated with tumor histologic grade and lymph mode metastasis and therefore is also considered an important marker in the evaluation of tumor cell proliferative activity [18, 21, 22]. The cancer suppressor gene p53 is very susceptible to mutation in tumors and plays a critical role in cell growth and regulation [23]. Positive expression of Ki-67 and p53 reflects the ability of tumor cells to infiltrate and metastasize and thus has been related to a relatively poor prognosis.

Angiogenesis is essential for tumor cell growth and infiltration and is the basis of CEUS [24]. Pathophysiologically, some tumor cells produce large amounts of VEGF, thus generating many new tiny blood vessels [25]. However, the rate of division of malignant cells is so high that the supply of nutrients by newly formed blood vessels is soon outstripped. This may explain why our Group 2 samples evinced more heterogeneous enhancement, including perfusion defects and peripheral enhancement. In our study, the comparison of enhancement pattern parameters between Group 1 and Group 2 showed statistically significant differences (*P* < 0.05). In accordance with these findings, previous studies have suggested that peripheral rim enhancement on MRI is associated with larger tumor size, higher histologic grade, ER-negative status, PR-negative status, and positive lymph node status [26, 27].

We found an absence of penetrating vessels more commonly in ER-negative tumors in Group 2. A previous study has reported that penetrating vessels were a potential means for differentiating between benign and malignant breast lesions and were significantly correlated with prognosis [28]. However, in our study, the absence of penetrating vessels was seen in Group 2 breast CEUS and correlated with ER negativity. Histologic analysis of ER-negative breast cancer demonstrated that ER-negative tumors commonly show signs of central necrosis or fibrosis [29]. It is possible that such necrosis could explain the absence of penetrating vessels.

Our results show a higher ratio of centripetal enhancement and absence of penetrating vessels to VEGF negativity in tumors >20 mm in diameter. Bzyl et al. [30] reported that very small lesions show higher binding of VEGFR2-specific microbubbles than larger tumors in their experiment (*P* < 0.023). Liu et al. [31] reported that VEGF was expressed peripherally while being centrally negative in tumors that showed peripheral enhancement. We would suggest that, with the rapid growth of tumors, the vascular formation and delivery of nutrition are relatively insufficient; thus, the center of the lesions may become hypoxic and necrotic, reducing the expression of VEGF and changing the distribution of microvessels.

The enlargement of enhancement area reflected that the breast cancers were abundant of angiogenesis. In our results, enlargement of the enhancement area was significantly associated with ER expression in Group 1 (maximum diameter ≤20 mm). Previous work showed that the region of size increased at CEUS was a sign in the diagnosis of malignant lesions [32]. For this group, lesions showing enlargement of enhancement area and homogeneous enhancement on CEUS may be due to a balanced spatial distribution of tumor blood vessels and small lesion size. Another possible explanation may be the high degree differentiation of early breast cancers. Early detection is crucial for diagnosis, treatment, and prognosis and the CEUS feature may be conductive to the diagnosis and prognosis assessment of early breast cancer.

Our study had some limitations. First, CEUS quantitative analysis and the time-intensity curve (which may be obtained from the region of interest by special software) were not assessed. Some researchers, however, have reported that the ability of CEUS qualitative analysis to identify breast tumors is preferable to quantitative analysis [33]. Second, only the maximum plane including the whole lesion and its surrounding normal tissue was selected for CEUS. It might be argued that it would have been better to study the lesion in its entirety. Third, axillary lymph node status, an important prognostic factor used to predict breast cancer recurrence and overall survival, was not assessed in this study. Finally, the sample size was relatively small, and further studies on larger populations are needed to confirm the results.

## 5. Conclusions

In conclusion, breast cancers with different sizes show different CEUS features and were conducive to the prognosis assessment of the breast malignances. What is more, CEUS could reduce unnecessary biopsies or reviews and it might be used as a prognostic reference. Maybe in the future we can use the CEUS characterizations as a clue to guide treatment protocols for breast cancers with different sizes.

## Figures and Tables

**Table 1:** CEUS features in Groups 1 and 2.

CEUS features	Total (*n*)	Group 1 (*n*)	Group 2 (*n*)	*P* value
Enhancement degree	104			0.985
Hyperenhancement		38	46	
Iso- or hypoenhancement		9	11	
Enhancement pattern	104			<0.001
Homogeneous		36	20	
Heterogeneous		11	37	
Penetrating vessels	104			0.486
Present		23	24	
Absent		24	33	
Enhancement order	104			0.479
Centripetal		32	35	
Centrifugal/diffuse		15	22	
Enhancement area	104			0.844
Enlargement		33	39	
Nonenlargement		14	18	

**Table 2:** Distribution of CEUS enhancement patterns and prognostic factors.

CEUS performance	ER^G1^		ER^G2^		PR^G1^		PR^G2^		Ki-67^G1^		Ki-67^G2^		P53^G1^		P53^G2^		c-er-B2^G1^		c-er-B2^G2^		VEGF^G1^		VEGF^G2^	
	+	−	+	−	+	−	+	−	+	−	+	−	+	−	+	−	+	−	+	−	+	−	+	−
Enhancement degree																								
Hyperenhancement	28	10	29	17	26	12	26	20	24	14	35	11	30	8	38	8	20	19	31	20	21	17	19	27
Iso- or hypoenhancement	6	3	6	5	6	3	6	5	4	5	7	4	5	4	8	3	1	7	1	5	6	3	7	4
Enhancement order																								
Centripetal	20	12	24	11	21	11	12	13	17	15	26	9	23	9	29	6	17	15	20	15	19	13	12	23
Centrifugal or diffuse	14	1	11	11	11	4	10	22	11	4	16	6	12	3	17	5	4	11	12	10	8	7	14	8
Penetrating vessels																								
Present	17	6	19	5	16	7	17	7	15	8	20	4	17	6	20	4	9	13	16	8	15	8	15	9
Absent	17	7	16	17	16	8	15	18	13	11	22	11	18	6	26	7	13	13	16	17	12	12	11	22
Enhancement pattern																								
Heterogeneous	6	5	21	16	7	4	20	17	6	5	28	9	6	5	29	8	10	12	19	16	5	6	14	23
Homogeneous	28	8	14	6	25	11	12	8	22	14	14	6	29	7	17	3	11	14	13	9	22	14	12	8
Enhancement area																								
Enlargement	27	6	25	14	23	10	22	17	21	12	30	9	26	7	32	7	17	16	25	14	21	12	19	20
Nonenlargement	7	7	10	8	9	5	10	8	7	7	12	6	9	5	14	4	4	10	7	11	6	8	7	11

Note: data are the number of cases. G1: Group 1; G2: Group 2.

**Table 3:** Association between CEUS characteristics and prognostic factors in the two groups.

	ER^G1^	ER^G2^	PR^G1^	PR^G2^	Ki-67^G1^	Ki-67^G2^	P53^G1^	P53^G2^	c-er-B2^G1^	c-er-B2^G2^	VEGF^G1^	VEGF^G2^
Enhancement degree	0.672	0.603	0.919	0.906	0.304	0.400	0.148	0.456	0.059	0.039	0.534	0.182
Enhancement order	0.028	0.161	0.597	0.197	0.188	0.897	0.552	0.603	0.121	0.847	0.696	0.030
Enhancement pattern	0.132	0.327	0.718	0.666	0.698	0.642	0.083	0.545	0.920	0.722	0.358	0.109
Penetrating vessels	0.813	0.019	0.831	0.057	0.440	0.158	0.932	0.668	0.770	0.172	0.292	0.029
Enhancement area	0.026	0.538	0.716	0.952	0.384	0.414	0.297	0.704	0.205	0.075	0.188	0.489

Note: data are *P* values (*χ* ^2^ test). G1: Group 1; G2: Group 2.

**Table 4:** The results of logistic regression analysis.

Independent variable	B	SE	Wals	*P*	Exp
ER^G1^					
Enhancement area	1.504	0.700	4.622	0.032	4.500
Constant	−0.154	0.556	0.077	0.782	0.857
ER^G2^					
Penetrating vessels	1.396	0.612	5.209	0.022	4.037
Constant	−1.224	0.509	5.786	0.016	0.294
VEGF^G2^					
Enhancement order	−1.210	0.569	4.531	0.033	0.298
Constant	1.056	0.410	6.620	0.010	2.857
VEGF^G2^					
Penetrating vessels	1.396	0.612	5.209	0.022	4.037
Constant	−1.224	0.509	5.786	0.016	0.294

Note: G1: Group 1; G2: Group 2.
